# Detection of a pederin‐like compound using a dilution‐to‐extinction‐based platform for the isolation of marine bacteria in drug discovery strategies

**DOI:** 10.1111/1751-7915.13679

**Published:** 2020-10-23

**Authors:** Xulio Benítez, Elena G. Gonzalez, Jesus García, Paz Zúñiga, Fernando de la Calle, Carmen Cuevas

**Affiliations:** ^1^ Research and Development Area PharmaMar S.A. Avda. De los Reyes, 1 P.I. La Mina‐Norte Madrid Colmenar Viejo 28770 Spain

## Abstract

The continued development of culturing technologies for the discovery of new molecules from marine microbes is of paramount importance for drug discovery. Coupled with this, the use of the high‐throughput approach shows promise for increasing the number of Gram‐negative and non‐filamentous bacteria cultures that can be surveyed, since they show a lower potential of bioactivity. In this work, we propose a new strategy of high‐throughput cultivation of bacteria inspired by a dilution‐to‐extinction (DTE) methodology for the isolation of, and screening for, new cytotoxic compound producing marine bacteria. A marine sponge tissue was directly used as inoculum and the results were compared with the data obtained through the direct plating isolation method. Enterobacterial repetitive intergenic consensus polymerase chain reaction (ERIC‐PCR) genomic fingerprinting indicated the isolation of four bioactive strains, three of them producers of a pederin‐like compound, and the fourth one able to synthesize a different compound, still unidentified, rendered by the DTE approach, in comparison with one bioactive strain identified through the plating method. Analyses based on the 16*S* rRNA gene data showed the existence of two different species belonging to the genus *Labrenzia*. The efficiency and diversity ratio in the number of isolates and compounds are discussed. In view of the results, the proposed DTE approach proved to be efficient for the isolation of new cytotoxic compounds of marine origin and pave the way for future potential applications.

## Graphical abstract

A new strategy of high‐throughput cultivation of bacteria inspired by a Dilution‐to‐Extinction methodology for the isolation and screening of new cytotoxic compound‐producing marine bacteria. A marine sponge tissue was directly used as inoculum and the results were compared with the data obtained through the direct plating isolation method. Results validate the combination of DTE with a 384‐plate format and antiproliferative tests for the exploration of cultures for mew marine strains and pave the way for future potential applications.

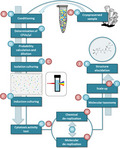

## Introduction

Since ‘The Great Plate Count Anomaly’ was put forth by Staley and Konopka (Staley and Konopka, 1985), a vast effort has been made to minimize the disparity between the immense bacterial diversity and abundance detected in samples by microscopy or metagenomics and the low number of isolates cultured. A large part of the environmental microbiome has been considered non‐cultivable using standard cultivation techniques or common condition media. However, there is a potentially number of species that can be cultured under other specific conditions (Keller and Zengler, 2004; Stewart, 2012; Overmann *et al*., 2017). These microorganisms could constitute an outstanding untapped source of pharmaceutically relevant organisms (Romano *et al*., 2017). With respect to the marine environment, this is even more evident, containing a large diversity of unusual natural products still unexplored (Alves *et al*., 2018; Nigam *et al*., 2019; Jimenez *et al*., 2020). Hence, the continued development of culturing technologies for the discovery of new molecules from marine microbes is of paramount importance for drug discovery (Klein Jan *et al*., 2017; Carroll *et al*., 2019).

There are relevant examples that claim an important role of non‐filamentous marine bacteria as synthesizers of potent cytotoxic compounds (Piel, 2009). In addition, despite the importance of the genus *Streptomyces* and other actinomycetes among marine bacteria with the ability to synthesize secondary metabolites (Dias *et al*., 2012; Zhang *et al*., 2017), genomic mining studies have shown an unexpected potential for providing insights into bioactive compound synthesis in Gram‐negative bacteria (Machado *et al*., 2015). A recent example has been found in our own research group, with the isolation of an alphaproteobacteria with the ability to synthesize a powerful bioactive compound previously considered to be of a macro‐organismal origin. The discovery of this *Labrenzia alexandrii* PHM005 as the producer of the new pederin‐like compound, labrenzin (Schleissner *et al*., 2017), supports the broadly accepted idea of a microbial origin of most natural products isolated from marine macro‐organisms (Piel, 2009).

Moreover, it had been thought that the producers of most natural products isolated from marine macro‐organisms are often microbiologic uncultivable symbionts, and findings like this run contrary to that idea. These discoveries have encouraged us to include non‐filamentous bacteria in the core of our high‐throughput screening programmes and to explore the different options for their isolation.

From easy‐to‐use methods like solid media plating, to sophisticated and complex ones such as the microencapsulation technique (Zengler *et al*., 2002), many achievements were reached (Stewart, 2012). Isolation by plating samples on solid media is the most widely used method (Joint *et al*., 2010). The minimal amount of equipment needed and the unlimited number of different culture media maintain solid media plating as the preferred isolation strategy for a wide range of bacteria. However, inhibition and competition phenomena, scalability, morphology‐guided selection or automation are just some limitations to overcome. Notwithstanding this, several different approaches have been applied successfully. Isolation techniques like I‐tip (Jung *et al*., 2014) and I‐chip (Nichols *et al*., 2010) are alternative options for mimicking the natural environment, including the marine habitat. Both methods allow the diffusion of natural signalling compounds, favouring the growth of microorganisms on their own environment. Single‐cell isolation methods such as microencapsulation (Ben‐Dov *et al*., 2009) or the use of the Microdish^®^ system (Ingham *et al*., 2007) have taken a step forward in mimicking in the laboratory the marine environmental conditions of the samples, growing pure cultures by individualizing single cells from their neighbours but keeping them connected to the outside environment and allowing diffusion in both directions of small signalling molecules. Nonetheless, scalability and high throughput are limiting factors for all the above‐mentioned techniques. Another single‐cell isolation technique is the dilution‐to‐extinction method (DTE) (Schut *et al*., 1993; Rappé *et al*., 2002), which has the feasibility of being easily scalable and automatable, allowing for very high‐throughput isolation (Stewart, 2012). This approach has proven to be very useful for the isolation of bacteria featuring special metabolic characteristics (Hoefman *et al*., 2012; Castro *et al*., 2017), such as methane‐oxidizing bacteria (Hoefman *et al*., 2012). More extensively, DTE has been successfully applied for the isolation of new marine oligotrophic bacteria throughout the last 30 years (Schut *et al*., 1993; Connon and Givannoni, 2002; Mohamed, 2013; Castro *et al*., 2017). These types of bacteria have several characteristics that make them less valuable for drug discovery programmes with respect to copiotrophical bacteria groups. Features such as small genome sizes or low growth rates are directly related to a reduced ability to produce secondary metabolites (Overmann *et al*., 2017). Thus, the DTE approach has been mainly focused on taxonomic studies, rather than producers of new and interesting secondary metabolites. In seeking innovative approaches for the discovery of new Gram‐negative bioactive producers, we realized that the lack of cytotoxic activity of most of its representatives, which keeps them outside the focus of high‐throughput screening programmes, could be an advantage for the implementation of an isolation and screening protocol based on DTE methodology. One of the main problems related to the use of *Actinomycetes* in drug discovery programmes is the high number of bioactive samples and the continuous rediscovery of existing compounds (Weber and Kim, 2016). The reduced number of bioactive samples obtained from non‐actinobacteria cultures (less than 2%, based on our experience), compared with those from *Streptomyces* (more than 30%, based on our experience), gave us the chance to set up a high‐throughput isolation platform combining DTE and antiproliferative assays. This allowed us to discard redundant and non‐cytotoxic activity samples in the first steps of the process.

**Fig. 1:**
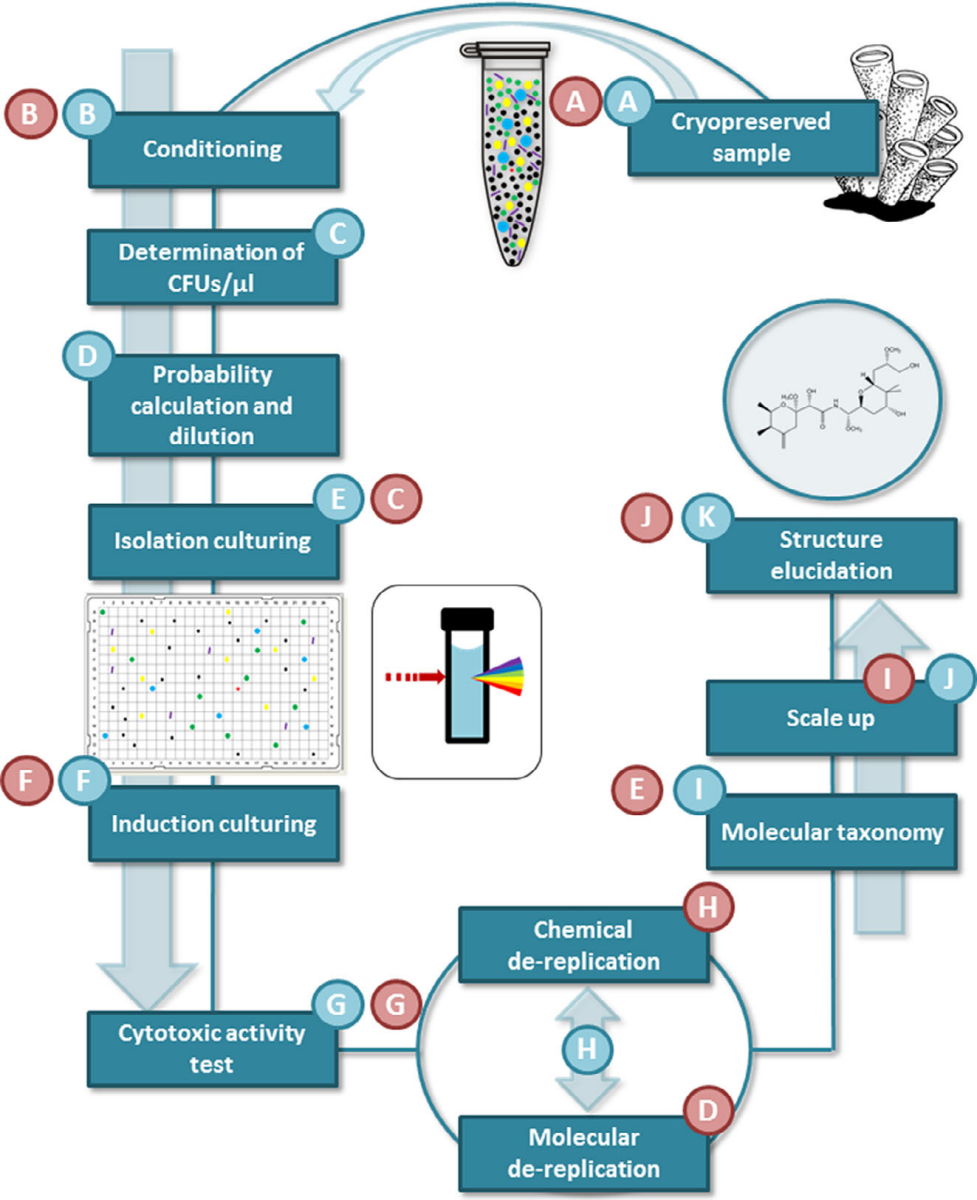
Flowchart of DTE methodology steps (blue letters), from the cryopreserved sample (A) to the new molecule structure elucidation (K). Red letters (A–J) were added in order to compare solid media with DTE isolation methods. While DTE goes directly from isolation culturing (E, in blue) to induction culturing (F, in blue), solid media isolation goes usually through molecular de‐replication and 16S rRNA gene sequence analysis steps (D and E, in red) to induction culturing step (F, in red).

Keeping this in mind, we intended to apply a DTE technique coupled with a high‐throughput platform (based on the use of 384 format well plates) as a complementary method to the classic solid media isolation, with the aim to discovering new marine bacteria with the ability to synthesize unrevealed cytotoxic compounds. Seeking innovative approaches for the discovery of new Gram‐negative producers, the classical workflow, sample, isolation, cultivation and screening, is modified by jumping directly from sample to cultivation, skipping the expensive, time‐consuming and repetitive isolation step (Fig. 1). The number of pure colony‐forming units (CFU) was statistically calculated by serially diluting the sample, and the obtained isolates were fermented for induction of secondary metabolite production. The crude extracts were tested in a bioassay against four cancer cell lines. Only samples with cytotoxic activity proceed to DNA–fingerprint and/or chemical de‐replication by HPLC‐MS, excluding a high number of strains with no interest for the isolation, re‐isolation and molecular and chemical de‐replication steps. Moreover, isolation cultures were carried out in 384‐well microtitre plates, a very appropriate format for automation. In addition, the fermentation volume used was small enough to allow the single cells to reach visible optical density in a short period of time (Fig. 1).

## Results

### Dilution calculation

**Fig. 2:**
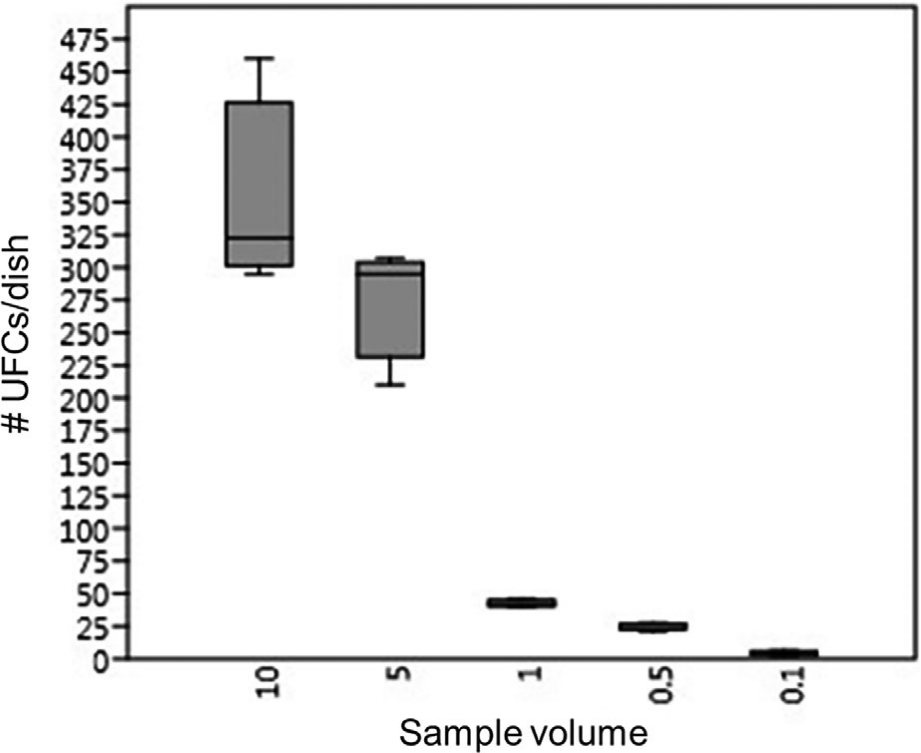
Box plot representation of the number of pure colony‐forming units (CFU) grown per plate using a volume of 10, 5, 1, 0.5 and 0.1 µl of the homogenized sample. Box shows median and interquartile range. Based on this information, the data for the 1, 0.5 and 0.1 µl inoculated volume were used for the dilution‐to‐extinction calculations.

The sample, a marine sponge, was homogenized, filtered and the mixture was serially diluted. A volume of the resulting aliquots was inoculated on solid media and incubated for 10 days at 28°C. Four replicates of each dilution were done and the grown colonies were counted daily in order to determine the cultivation parameters and statistical calculations. The average CFUs µl^−1^ for the homogenized and filtered sample was established in 47.58, and thus, the calculated number of expected grown wells in each microtitre plate (384‐well) for a percentage of mixed cultures of 5% was 85.86. Strong inhibition phenomena and variance between replicates were observed on inoculated solid media with high sample volumes. The range tested for optimal growth rates concluded that densities below 50 CFUs per plate improved reproducibility and proportionality between replicates and inoculated volumes. In this sample, that density was obtained with the plates inoculated with 1, 0.5 and 0.1 µl of homogenized sample (Fig. 2). Nevertheless, when the sample was inoculated on liquid media and fermented in 384 well microtitre plates, the number of grown wells was twofold more than expected. Thus, a correction factor was applied to offset the growth rate in the transition from solid to liquid media.

### Isolation and screening of cultures on 384‐well microtiter plates based on the cytotoxic activity

Fifty microtiter plates were inoculated with the calculated dilution. Four days after, 2242 wells with potential bacterial growth were detected by optical density measurement. To avoid overgrowth and death of the fastest growing cultures, the growth wells were selected, inoculated onto 96‐well plates and incubated again with fresh Marine Broth (Difco^®^ 2216) for 96 h. The 81.5% of the cultures grew in this step (based on the optical density measurement). They were inoculated in the culture media (2MPD1 F), and after 5 days, each culture was lyophilized, extracted and tested against four human cancer cell lines. The cytotoxic assay gave a total yield of 28 samples with cytotoxic activity (Table S1).

### Molecular analyses of the positive cultures

**Fig. 3:**
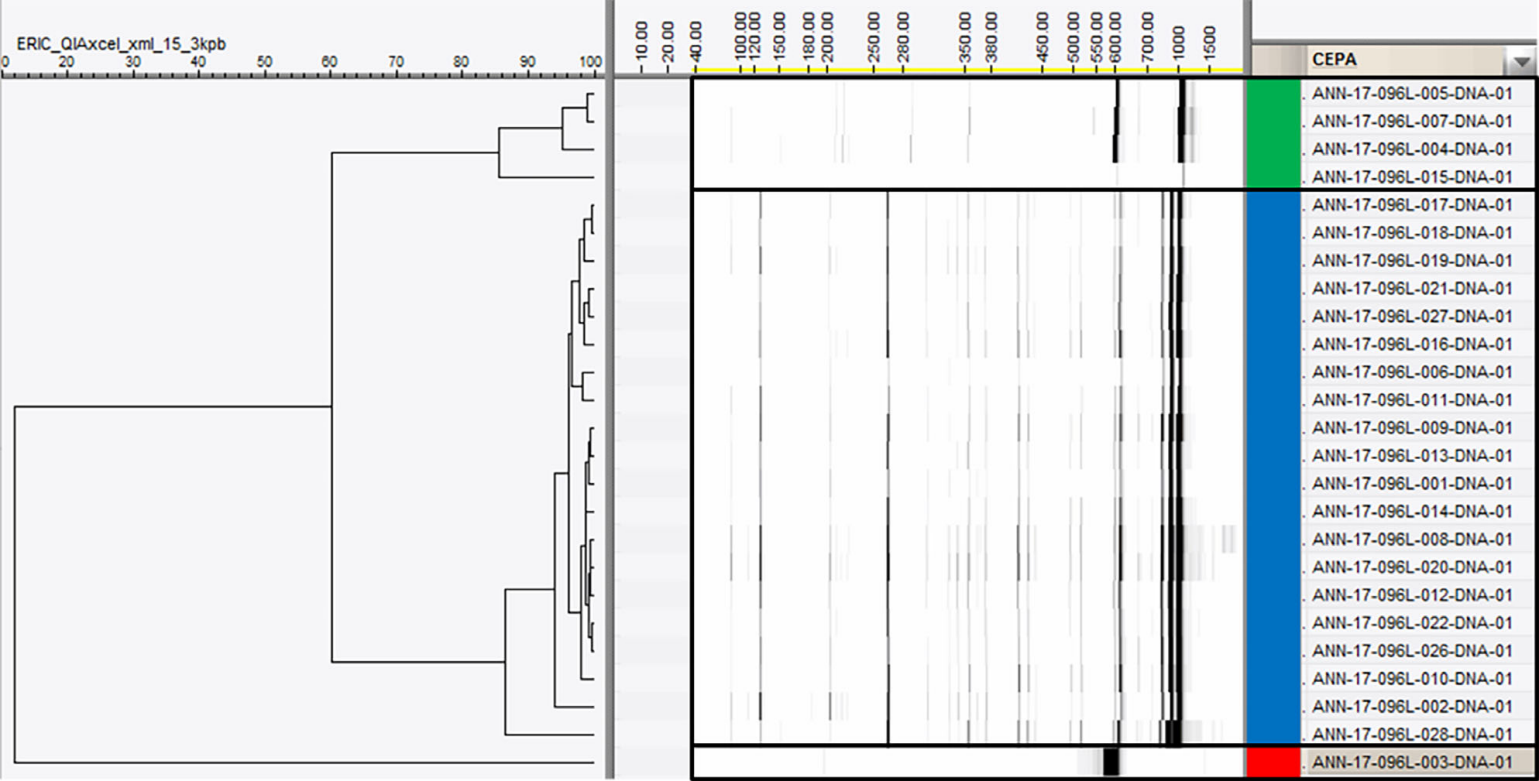
Genetic fingerprint results of the 25 active pure cultures isolated with DTE. The strains were divided into 3 different groups showing similar isolates among them: I (green bar), II (blue bar) and III (red bar). Strains from groups I and II were identified as the species *Labrenzia alexandrii* and the only one from group III was identified as a *L. aggregata,* based on their 16*S* rRNA gene sequence analyses. The scale bar indicates percentage of similarity.

**Fig. 4:**
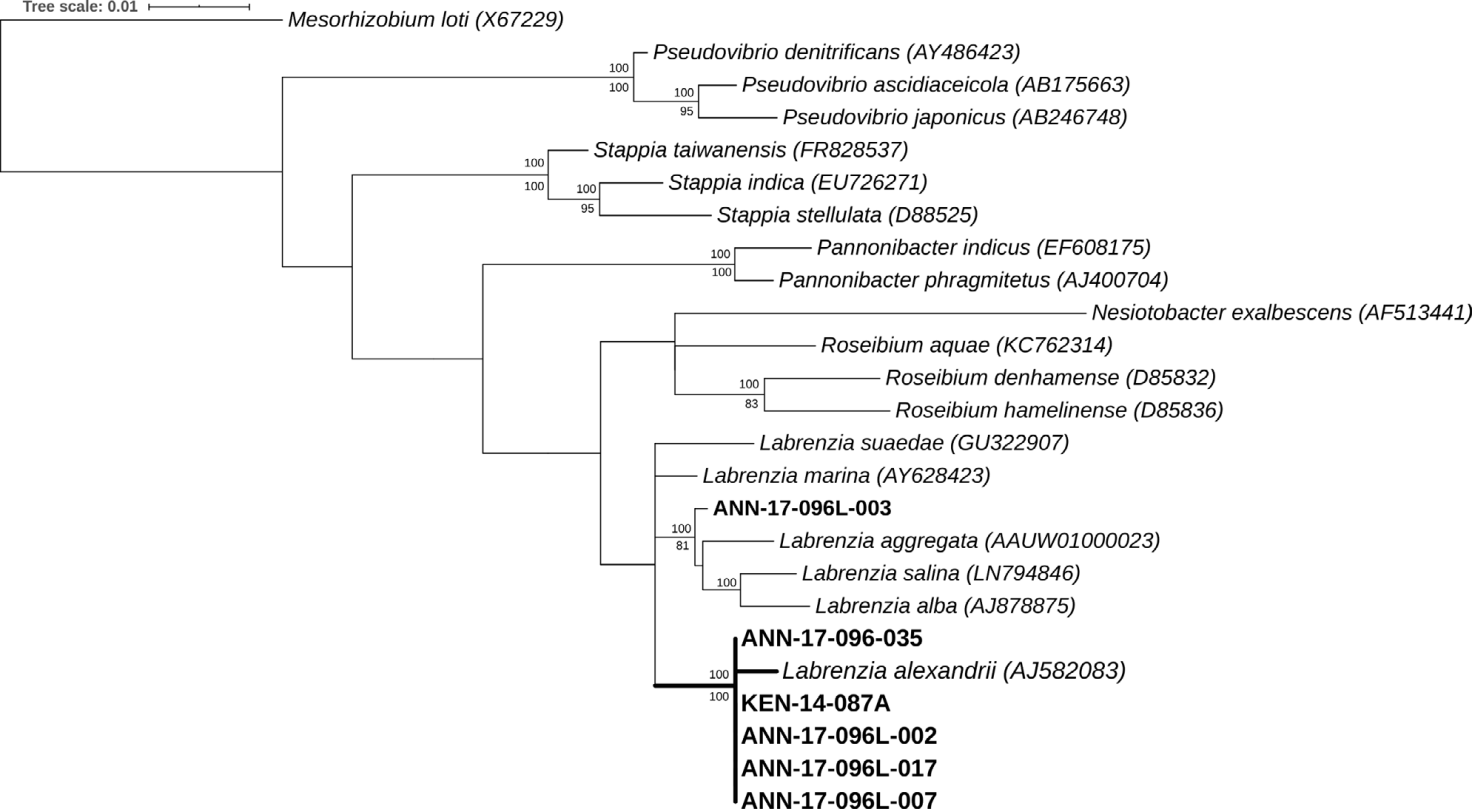
Neighbour‐joining (NJ) and maximum‐likelihood (ML) tree of 16*S* rRNA gene sequences reconstructed using TIM distances. In bold, the samples sequenced for this work are included. The SILVA Accession numbers are indicated in brackets. Bootstrap values are from ML (above node) and NJ (below node) analyses. The tree is rooted with *Mesorhizobium loti*. SILVA accession numbers accompany each taxon name. The scale bar indicates evolutionary distances.

The 28 active cultures were inoculated on solid media, obtaining 25 pure cultures, 3 showed mixed cultures and were discarded. The REP‐PCR fingerprint grouped the samples into 3 different clusters with 20, 4 and 1 representatives respectively (Fig. 3). Several strains from each cluster were later identified by PCR amplification, sequencing and phylogenetic analyses of a variable region of the 16*S* rRNA. The comparison of the sequences against the SILVA LTPs 132 database (http://www.arb‐silva.de/projects/living‐tree/) identified all the strains as the genus *Labrenzia,* with a high similarity value (> 99%). According to this result, both NJ and ML trees obtained from the phylogenetic analyses resulted in the same topology and yielded a well‐supported monophyletic *Labrenzia alexandrii* clade (Fig. 4). Most genera are highly supported, being monophyletic. Strain ANN‐17‐096L‐003 was more closely related to the *L. aggregata* (*AAUW01000023)* sequence than the *L. alexandrii* clade that included the positive strains obtained with the DTE method (Fig. 4), and was classified as such.

### Chemical de‐replication

The chemical de‐replication of the crude extract of one representative of each group determined the presence of the labrenzins (pederin analogues) PM160134 and PM160153 in 2 strains (Fig. S1).The third strain (named ANN‐17‐096L‐003, identified as *L. aggregata*) is in the process of elucidation.

## Discussion

The main aim of this study was to adapt the DTE methodology for the isolation of marine non‐filamentous bacteria with the final intention of detecting the ones with the ability to produce cytotoxic compounds (Machado *et al*., 2015). To explore this, a proof of concept was done using a marine sponge (*Haplosclerida* order) from where previously isolated pederin‐like molecules were detected (Schleissner *et al*., 2017). Another bacterial isolation technique, i.e. solid plate isolation technique, was applied in parallel for comparison purposes (Klein Jan *et al*., 2017). To our knowledge, this is the first study to apply DTE coupled with an automatizable 384‐liquid media format, followed by antiproliferative tests for human cancer cell lines, to a marine sponge‐associated bacteria, in order to look for new chemical entities to fight against cancer.

Results indicated that the DTE approach has been successfully applied for the isolation of bioactive compounds and shows promise for future high‐throughput optimizations for increasing the number of non‐filamentous bacteria cultures that can be surveyed.

The solid media isolation method shows obvious advantages. It allows the isolation of microorganisms in a plethora of media with very simple equipment (Overmann, 2013; Bonnet *et al*., 2020). Nevertheless, it has some limitations that the DTE method can reinforce, e.g. the difficulty in scalability and automatization of the Petri dish format. Furthermore, the microfermentations in 384‐well plates format make this protocol very suitable for liquid handling equipment and thus for high‐throughput screening programme (Betts and Baganz, 2006). Moreover, solid media isolation follows the sample‐isolation‐fermentation sequence, resulting in the isolation of a great number of redundant isolated bacteria (Fig. 1A–J in red). However, the tested method goes directly from sample to fermentation, and only bioactive cultures with novel compounds are isolated and characterized (Fig. 1A–K in blue), allowing to test thousands of strains for activity without plate spreading, colony visual picking, isolation and/or re‐isolation (Hoefman *et al*., 2012).

Another advantage observed of applying DTE cultivation, together with the 384‐liquid media format is the increase of culturability in terms of abundance of bacteria that could render in a higher biodiversity of species. For the DTE method, to determine the bacterial abundance of the sample needed for further dilution calculations, the count has been done indirectly by inoculating a volume of the homogenized sample on Petri plates filled with isolation agar medium instead of using other more common methods (e.g. microscope visualization or flow cytometry measure). Through this step, it was possible to avoid counting dead or non‐viable cells, allowing for a more accurate dilution calculation. By doing this, we observed that a high density of CFUs in the same Petri dish (over 50) induced inhibition or competition phenomena that inhibited the growth of some of the microorganisms, the number of CFUs ml^−1^ observed on the 384 microtiter plates being approximately twice that expected. These two issues indicate that DTE gets better results in terms of number of grown organisms than solid media isolation. Further studies would be needed to determine if the increase happens also in terms of biodiversity (Klein Jan *et al*., 2017). Moreover, the low 384‐well microculture volume used (50 µl) provides the bacteria with a comfortable environment for growing but still allows a single cell to reach detectable population densities. Truth being that some organisms need to grow together (Joint *et al*., 2010), it is also true that numerous microorganisms have the ability to produce chemical entities such as antibiotics that inhibit in some ways the growth of the closest individuals of other species. With the fermentation carried out in such a small volume and physically independent compartments, we prevent any kind of communication between cultures and obtain grown cultures after short periods of incubation. This conclusion is supported by twofold increase in recovered cells CFUs µl^−1^ recounted on liquid media 384‐well plates, with respect to solid media Petri dishes. In our assay, the percentage of mixed bioactive culture obtained (11%) were higher than the threshold established (5%) but still being admissible, taking into account different factors that affect the dilution, such as particles or consortia.

Regarding the diversity of taxa obtained, the fact that our DTE method has been able to isolate the producer *L. alexandrii* becomes relevant. Moreover, the finding of three bioactive strains of the same genus (and different species) in comparison with the bioactive strain obtained with the solid plate method is very significant. The bioactive strains isolated with the DTE method showed the same morphology and it would have been discarded if a colony visual picking selection were applied. In that sense, the isolation of different strains of the same species provides an opportunity to find better producers since they might synthesize different molecules (Romano *et al*., 2018) which would help in a hypothetical improvement of the production process. However, the expansion of the number of samples to be assayed with the DTE method in parallel with a broader morphological isolation of samples obtained on solid media is needed in order to have a more reliable biodiversity rate of cultivable bacteria.

Together, these results validate the combination of DTE with a 384‐plate format and antiproliferative tests for the exploration of cultures for mew marine strains. We are currently expanding the potential of DTE to the exploration of cultures for new marine strains by enabling the use of different media and isolation timings.

## Experimental procedures

### Sample collection and conditioning

The studied organism is a marine sponge belonging to the *Haplosclerida* order. It was collected in January 2017, from a depth of 47 metres, off the shores of Palau (02°55.657′N 131°47.592′E) in the Pacific Ocean. The sample was directly preserved at −30°C in water with glycerol (20% v/v) and sea salts (Tropic Marine PROREEF 2.7% w/v). In the laboratory, the sample was mechanically homogenized with mortar and filtered through an 80‐µm mesh to discard debris. The final volume was kept at −80°C in 200‐µl Eppendorf tubes (Fig. 1B in blue).

### Dilution‐to‐extinction calculations

To determine the average number of colony‐forming units per µl (CFUs µl^−1^) in the sample, inoculation of the homogenized sample on marine agar (Marine Broth, Difco^®^ 2216) with cycloheximide (0.02% w/v) was performed. It was carried out through four replicate dilutions of 10, 5, 1 and 0.1 µl of the homogenized previously suspended in a total volume of 100 µl of purified water with marine salt (27 g l^−1^) (Fig. 1C in blue). The dishes were incubated at 28°C during 2 weeks and the colonies were counted daily to determine the moment of no more colonies growth, which in our case, corresponded to ten days. The results after statistical treatment showed that means from 10 and 5 µl per plate were not reproducible because of their high standard deviation (Fig. 2). Hence, the CFUs µl^−1^ were calculated based on the 1, 0.5 and 0.1 µL dilution replicates, with a resulting average number of 47.6 CFUs µl^−1^ in the sample.

In order to maximize the number of pure cultures per plate with the lowest number of mixed cultures, a probability calculation of independent events was made based on *P* **_AB_** = *P*_A*_ *P*_B_, where *P* **_AB_** is the calculated probability and *P* **_A_** and *P* **_B_** the probability of each independent event. If the accepted percentage of twice inoculated wells is established in 5% (*P* **_AB_**) and the probability of each well of being inoculated is N/384 (for *P* **_A_** and *P* **_B_**); then, the number of calculated CFUs inoculated per plate (N) would be 85.86. For the next step of inoculation in liquid media, we corrected the volume of homogenized sample used as a factor for the higher number of colonies that grew in liquid, in comparison with solid media (Fig. 1D in blue).

### Isolation, grown culture picking and induction cultures

The broadly used medium for marine bacteria, marine broth (Difco 2216), supplemented with cycloheximide (0.02% w/v), was used for growing *L. alexandrii*, since it was reported to be adequate for that species (Sipkema *et al*., 2011). Moreover, its low absorbance made it suitable for the DTE isolate detection. Cultures were done in 384‐well polystyrene plates with a volume of 50 µl of marine broth per well. Filling and inoculation of plates were done by the liquid handling robot EVO100 (Tecan^®^, Männedorf, Switzerland). The progressive appearance of grown wells was determined, based on 5 replicates. The results showed a slowdown in the appearance of new CFU after 5 days of incubation. Overall, incubation was set to 5 days at 28°C and 280 r.p.m. in a rotatory shaker (Kuhner^®^, Basel, Switzerland) with 5 cm eccentricity. The detection of wells with growth was performed by optical density measurement, using an Omega POLARstar (BMG LABTECH^®^, Ortenberg, Germany) reader. The detection threshold was set over 0.3 at 600 nm (Fig. 1E in blue). The recovery of the cultures from the 384‐well plates and the inoculation on 96‐well plates with 750 µl of fresh medium Marine Broth (Difco^®^ 2216) per well, was performed with a liquid handling robot MicroLab Star (Hamilton^®^, Reno, NV, USA). The plates were incubated for 5 days at 28°C and 280 r.p.m. All the remaining volume, after inoculation of the induction cultures, was stored at −80°C with glycerol (20%) for further analyses. Finally, the induction cultures (Fig. 1F in blue) were carried out in the medium 2MPD1 F (soybean flour (Sigma^®^, Saint Louis, MO, USA) 5 g l^−1^, bacto peptone (Difco^®^, Hampton, NH, USA) 2 g l^−1^, corn steep powder (Roquette Solulys^®^ 095E) 2 g l^−1^, mannitol 20 g l^−1^, CO_3_Ca 10 g l^−1^, sea salts (Pro Reef^®^) 36 g l^−1^) and 3MTM F (soybean flour (Sigma^®^) 20 g l^−1^, corn steep powder (Roquette Solulys^®^ 095E) 5 g l^−1^, glycerol 5 g l^−1^, CO_3_Ca 10 g l, sea salts (Pro Reef^®^, Wartenberg, Germany) 20 g l^−1^) with 5 days of incubation at 28°C and shaking at 230 r.p.m.

### Extraction and antiproliferative experiments

After incubation, the broths were lyophilized and extracted with a mixture of methanol, acetone and water (1:1:0.2 v/v). The dried crude extracts were screened for cytotoxic activity (Fig. 1G in blue) against four tumour cell lines: A549 (ATCC CCL‐185) (lung carcinoma, NSCLC); HT‐29 (ATCC HTB‐38) (colon adenocarcinoma); MDA‐MB‐231 (ATCC HTB‐26) (breast adenocarcinoma); and PSN‐1 (ATCC CRL‐3211) (pancreas adenocarcinoma). All the cell lines derived from human cancer and were obtained from the American Type Culture Collection (ATCC). Cells were maintained in Dulbecco’s modified Eagle’s medium (DMEM) (except PSN‐1 that was maintained in Roswell Park Memorial Institute 1640 Medium (RPMI)), supplemented with 10% fetal bovine serum (FBS), 2 mM l‐glutamine, 100 U ml^−1^ penicillin and 100 U ml^−1^ streptomycin at 37°C, 5% CO_2_ and 98% humidity. For the experiments, cells were harvested from subconfluent cultures using trypsinization and resuspended in fresh medium before counting and plating. Crude extracts are resuspended with DMSO and DMEM and dispensed to culture plates containing the cells (final concentration of DMSO 1% (v/v)) to perform the cytotoxic assay. After 48 h of treatment, cytotoxic activity was measured based on the inhibition of cellular proliferation values in comparison with the control cells (Table S1).

### DNA_Fingerprint and taxonomy

The samples that showed antitumour activity were recovered from −80°C and re‐cultured on solid marine agar (Marine Broth, Difco^®^ 2216). Genomic DNA from pure isolates was extracted using the Qiagen DNeasy tissue kit (Qiagen). ERIC‐PCR genomic fingerprinting (Fig. 1H in blue) was performed using ERIC‐2 primer (5′‐AAG‐TAA‐GTG‐ACT‐GGG‐GTG‐AGC‐G‐3′), following the amplification conditions described in Versalovic *et al*. (1991). The bands generated by electrophoresis of the PCR amplifications were analysed in a QIAxcel (Qiagen), and sizes were calculated by comparison with DNA molecular size markers (100 bp–2.5 Kb marker, Qiagen). The fingerprint information from other bacteria strains of the genus *Labrenzia* that produce Labrenzin was also included in the analyses. Cluster analyses were performed in bionumerics software v7.6.3, employing the Pearson correlation coefficient with a 2% curve smoothing. A cut‐off value of 95% was used to separate genetically different isolates. From those that were identical isolates, the representative ones showing the higher cytotoxic activity were selected and included in the bacterial collection at PharmaMar. These samples were also subjected to 16*S* rRNA gene sequence analyses to determine their taxonomy based on BLASTp comparisons with SILVA LTP132 database (www.arb‐silva.de). Primers used for amplification were 63‐F (5′‐CAGGCCTAACACATGCAAGTC‐3′) and 1387‐R (5′‐GGGCGGWGTGTACAAGGC‐3′) (Marchesi *et al*., 1998). PCR was performed with an initial denaturation step at 95°C for 1 min, followed by 30 cycles of denaturation at 94°C for 1 min, annealing at 58°C for 50 s and extension at 72°C for 1 min, with a final extension step at 72°C for 10 min. Moreover, two strains of *L. alexandrii* (ANN‐17‐096‐035 and KEN‐14‐087A) from our collection were grown and sequenced for comparison purposes. Partial nucleotide sequences of the 16S rDNA gene were aligned with clustal X v2 using the default parameters and verified manually in order to maximize positional homology. All positions with gaps were omitted for the phylogenetic reconstructions. Additionally, sequences retrieved from the SILVA LTPS 132 database were also included in the analyses. The data set was subjected to the neighbour‐joining (Saitou and Nei, 1987) and maximum‐likelihood (ML) method of phylogenetic inference. Analyses were carried out with paup v4.0b10 (Swofford, 2003). The Akaike information criterion (AIC) implemented in modeltest v2 (Posada and Crandall, 1998) was used to select the evolutionary model that best‐fitted the empirical data set. The TIM (‘transitional model’) was selected as the best‐fitted model to the data set. Robustness of the resulting tree was tested with 1000 bootstrapping (Felsenstein, 1985).

### Chemical de‐replication

The remaining aliquots were further subjected to de‐replication through their mass spectra to look for known compounds (Fig. 1H). (+)‐ESIMS were recorded using an Agilent 1100 Series LC/MSD spectrometer. High‐resolution mass spectroscopy (HRMS) was performed on an Agilent 6230 TOF LC/MS system using the ESIMS technique.

### Solid media isolation and characterization of the sample

Solid media isolation of the sample was carried out in parallel to the previous method. Five different media, Benedict agar BEN (1/2 dilution), ammonium mineral salts (AMS), marine broth (Difco 2216), marine broth (Difco 2216) supplemented with streptomycin (100 mg l^−1^) and marine broth (Difco 2216) supplemented with trimethoprim (30 mg l^−1^) were used. Three replica plates of each condition were inoculated with 30 µl of the homogenized sample and incubated for ten days at 28°C. After incubation, visually different colonies were selected and re‐cultured in new plates to obtain pure growth. The characterization, fermentation and cytotoxic test of all the isolated bacteria were performed following the procedures described before, with some variations in the organization of the steps (Fig. 1A–J in red).

As a result, 35 morphologically different colonies were selected, and at the end of the process, one culture with a cytotoxicity assay‐positive result was detected. The active organism (named ANN‐17‐096‐035, Figs 3 and 4) was identified as *L. alexandrii*, and the cytotoxic activity was produced by the pederin‐like molecule, labrenzin.

## Conflict of interest

The authors declare no conflict of interest.

## Supporting information


**Fig. S1.** Chromatographic profile and mass spectra of the strain ANN‐17‐096L‐007 highlighting labrenzin characteristic peaks. The authenticity of labrenzin was inferred from its HRESI and its 2D NMR spectra previously described (Schleissner *et al*., 2017)

**Table S1.** Cytotoxic activity (measured as growth inhibition, in %) for the 28 active cultures obtained against the cancer cell lines. The threshold for antitumor activity was established for inhibition values below 25% in three of the four cancer cell lines tested.
