# Supplementary figures and images for: Detection of a pederin‐like compound using a dilution‐to‐extinction‐based platform for the isolation of marine bacteria in drug discovery strategies
Source: Microb Biotechnol. 2020 Oct 23;14(1):241–50. doi: 10.1111/1751-7915.13679 (PMC7888454; doi:10.1111/1751-7915.13679)

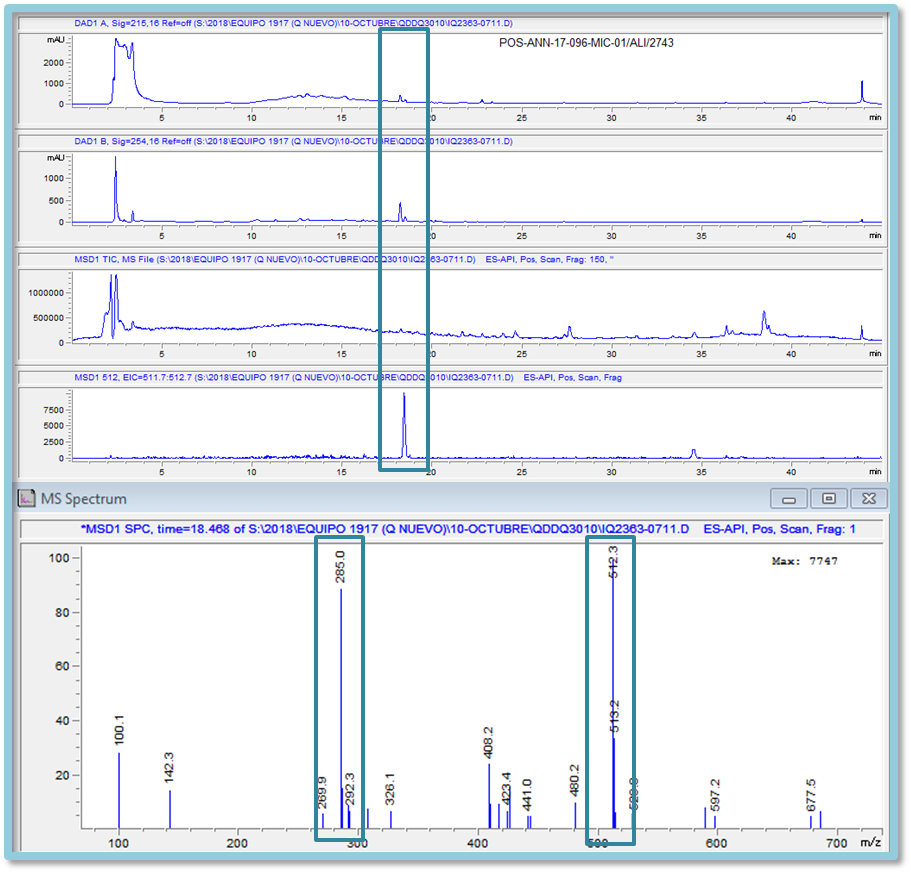

Supplement: Supplementary file 1 — Fig. S1. Chromatographic profile and mass spectra of the strain ANN‐17‐096L‐007 highlighting labrenzin characteristic peaks. The authenticity of labrenzin was inferred from its HRESI and its 2D NMR spectra previously described (Schleissner et al., 2017) [file MBT2-14-241-s001.tif]
